# 蛋白质*N*-磷酸化修饰富集方法进展

**DOI:** 10.3724/SP.J.1123.2024.04029

**Published:** 2024-07-08

**Authors:** Bo JIANG, Bo GAO, Shuxian WEI, Zhen LIANG, Lihua ZHANG, Yukui ZHANG

**Affiliations:** 1.中国科学院大连化学物理研究所,医学蛋白质组全国重点实验室,国家色谱研究中心, 中国科学院分离分析化学重点实验室,辽宁 大连 116023; 1. State Key Laboratory of Medical Proteomics, National Chromatographic R.& A. Center, CAS Key Laboratory of Separation Science for Analytical Chemistry, Dalian Institute of Chemical Physics, Chinese Academy of Sciences, Dalian 116023, China; 2.中国石油大学(华东)化学化工学院,山东 青岛 266580; 2. School of Chemistry and Chemical Engineering, China University of Petroleum (East China), Qingdao 266580, China

**Keywords:** 蛋白质*N*-磷酸化, 生物功能, 富集方法, 综述, protein *N*-phosphorylation, biological function, enrichment method, review

## Abstract

蛋白质磷酸化作为一种最普遍和最重要的翻译后修饰调控着几乎所有的生命过程。随着高效富集方法和生物质谱技术的快速发展,低丰度的蛋白质*O*-磷酸化修饰获得了规模化鉴定,从而使其生物学功能得到较为透彻的研究。而发生在组氨酸、赖氨酸和精氨酸侧链氨基的*N*-磷酸化修饰,由于P-N键化学稳定性差,导致其在酸和热条件下不稳定。而目前依赖酸性条件的*O*-磷酸化富集方法难以适用*N*-磷酸化富集,导致蛋白质*N*-磷酸化生物功能研究严重滞后。因此,迫切需要发展针对蛋白质*N*-磷酸化的高效富集方法。本文首先介绍了蛋白质*N*-磷酸化的结构特征和已报道的生物学功能,重点综述并分析了近20年来蛋白质*N*-磷酸化修饰富集方法,并对每一种富集方法的优缺点进行了评述,最后对潜在的富集方法进行了展望。

## Abstract (en)

Protein phosphorylation is one of the most common and important post-translational modifications that regulates almost all life processes. In particular, protein phosphorylation regulates the development of major diseases such as tumors, neurodegenerative diseases, and diabetes. For example, excessive phosphorylation of Tau protein can cause neurofibrillary tangles, leading to Alzheimer’s disease. Therefore, large-scale methods for identifying protein phosphorylation must be developed. Rapid developmentin efficient enrichment methods and biological mass spectrometry technologies have enabled the large-scale identification of low-abundance protein *O*-phosphorylation modifications in, allowing for a more thorough study of their biological functions. The *N*-phosphorylation modifications that occur on the side-chain amino groups of histidine, arginine, and lysine have recently received increased attention. For example, the biological function of histidine phosphorylation in prokaryotes has been well studied; this type of modification regulates signal transduction and sugar metabolism. Two mammalian pHis kinases (NME1 and NME2) and three pHis phosphatases (PHPT1, LHPP, and PGAM5) have been successfully identified using various biological methods. *N*-Phosphorylation is involved in multiple biological processes, and its functions cannot be ignored. However, *N*-phosphorylation is unstable under acidic and thermal conditions owing to the poor chemical stability of the P-N bond. Unfortunately, the current *O*-phosphorylation enrichment method, which relies on acidic conditions, is unsuitable for *N*-phosphorylation enrichment, resulting in a serious lag in the large-scale identification of protein *N*-phosphorylation. The lack of enrichment methods has also seriously hindered studies on the biological functions of *N*-phosphorylation. Therefore, the development of efficient enrichment methods that target protein *N*-phosphorylation is an urgent undertaking. Research on *N*-phosphorylation proteome enrichment methods is limited, hindering functional research. Thus, summarizing such methods is necessary to promote further functional research. This article introduces the structural characteristics and reported biological functions of protein *N*-phosphorylation, reviews the protein *N*-phosphorylation modification enrichment methods developed over the past two decades, and analyzes the advantages and disadvantages of each method. In this study, both antibody-based and nonantibody-dependent methods are described in detail. Owing to the stability of the molecular structure of histidine, the antibody method is currently limited to histidine phosphorylation enrichment research. Future studies will focus on the development of new enrichment ligands. Moreover, research on ligands will promote studies on other nonconventional phosphorylation targets, such as two acyl-phosphates (pAsp, pGlu) and *S*-phosphate (pCys). In summary, this review provides a detailed analysis of the history and development directions of *N*-phosphorylation enrichment methods.

蛋白质是生命功能的重要调控者,在翻译过程中蛋白质可被小分子共价修饰,形成蛋白质翻译后修饰^[1]^。蛋白质翻译后修饰通过改变蛋白质结构和化学性质来影响其定位、活性及相互作用,是生命活动的重要调控开关^[2]^。蛋白质磷酸化是最重要和最普遍的一类翻译后修饰,目前在原核生物和真核动物中均发现其存在。蛋白质磷酸化是指在激酶作用下,由三磷酸腺苷(ATP)提供磷酸根和能量将磷酸根共价键合到蛋白质残基的过程,而去磷酸化则是由磷酸酶催化的水解反应^[3]^。目前约有500种蛋白激酶和少量的磷酸酶调节着可逆的磷酸化过程。据统计,在哺乳动物细胞整个生命过程内约有1/3的蛋白质以磷酸化形式存在^[4]^。总之,蛋白质磷酸化与生命过程密切相关。

蛋白质分子中有9种氨基酸残基可以发生磷酸化修饰,根据发生磷酸化修饰的氨基酸类型的不同,可将磷酸化分为以下4种类型:(1)磷酸化修饰发生在丝氨酸、苏氨酸和酪氨酸的侧链羟基(pSer、pThr和pTyr),以P-O键连接的磷酰酯类修饰,被称为*O*-磷酸化^[5]^。丝氨酸是蛋白质中最常见发生*O*-磷酸化修饰的氨基酸,其次是苏氨酸,在已知的500多种激酶中,有125个是丝氨酸/苏氨酸磷酸化激酶。由于*O*-磷酸化性质稳定且研究透彻,被称为典型性磷酸化^[6]^。(2)磷酸化修饰发生在半胱氨酸侧链巯基,形成P-S键,称为*S*-磷酸化(pCys)。pCys最初被发现作为磷酸烯醇丙酮酸依赖性磷酸转移酶系统(PTS)的中间体^[7]^。(3)磷酸化修饰发生在天冬氨酸和谷氨酸的侧链羧基(pAsn和pGlu),形成P-OCO键,称为羧基磷酸化。研究表明pAsn被发现作为双组分组氨酸激酶信号系统的中间体,将来自组氨酸激酶的信号传递给DNA,并与转录因子共同参与启动基因的转录^[8]^。(4)磷酸化修饰发生在组氨酸、赖氨酸和精氨酸的侧链氨基(pHis、pLys和pArg),以P-N键连接的磷酰胺类修饰,称为*N*-磷酸化(图1)^[9]^。此外,根据磷酸化在组氨酸中的位置不同,又可以分为3-组氨酸磷酸化(3-pHis,热力学稳定产物)和1-组氨酸磷酸化(1-pHis,动力学稳定产物)^[10]^。除*O*-磷酸化外,其余3类磷酸化研究较少,被称为非典型性磷酸化^[11]^。蛋白质的可逆磷酸化过程在生命活动中起着重要的调控作用,蛋白质*O*-磷酸化的生物学功能得到系统和深入的研究。如Tau蛋白的过度磷酸化会导致神经纤维缠结,引起神经元细胞功能失调,进而导致阿兹海默病的发生和发展^[12]^。蛋白质磷酸化修饰也被证明参与调控神经细胞溶酶体自噬功能。例如,研究发现STK11IP是蛋白激酶mTORC1在溶酶体中的特异性底物。STK11IP通过丝氨酸磷酸化(pSer404)调控与V-ATPase的结合力,进而影响溶酶体自噬功能^[13]^。此外,PAK4或者SKA3可以直接磷酸化p53蛋白Ser215,从而抑制p53的转录活性和p53介导的抑制肝癌细胞侵袭的能力^[14]^。近年来随着抗体和生物质谱等技术的快速发展,蛋白质*N*-磷酸化的生物功能也逐步得到揭示。

**图1:**
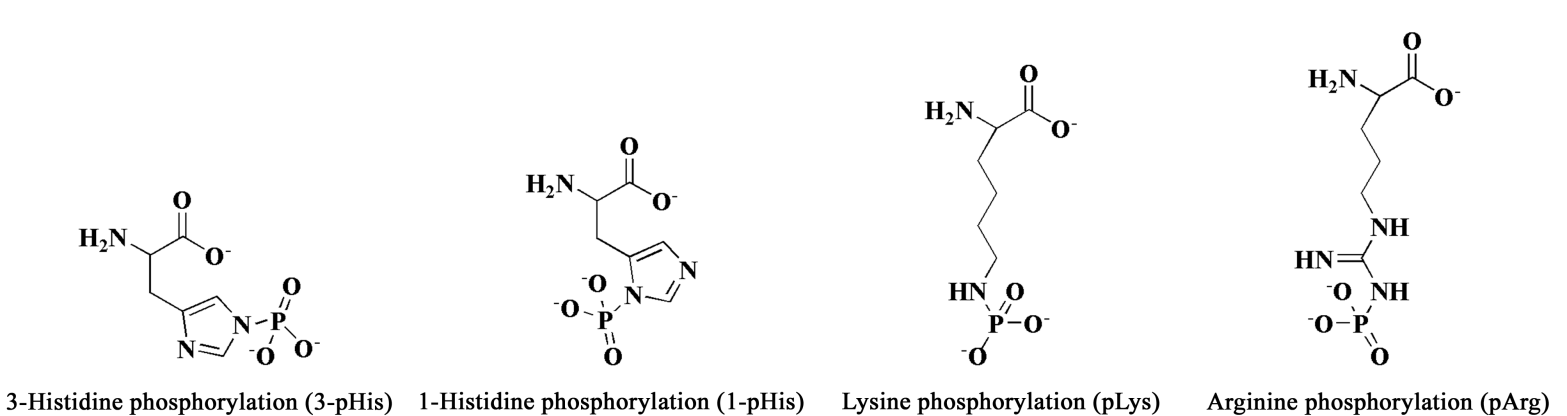
*N*-磷酸化结构示意图

## 1 蛋白质*N*-磷酸化的重要生物功能

### 1.1 pHis的生物功能

同*O*-磷酸化修饰相比,pHis功能研究相对滞后,研究表明生物体内pHis丰度与pTyr类似。1962年就有报道称牛肝脏线粒体蛋白质中存在pHis^[15]^,之后的研究表明pHis主要以激酶、磷酸酶和酶催化中间产物的形式存在^[16]^。在原核生物和低等真核生物中pHis蛋白质是细胞信号转导的重要调控开关^[17]^。例如,pHis通过双/多组分系统在细胞信号传导中发挥关键作用。细菌通过该系统感受外界环境变化来维持自身生存,是细菌适应选择压力的一种机制。该系统包括用于感应输入信号的pHis激酶感应器和用于调节输出信号的应答调控器。pHis激酶通常位于细胞质膜,用于感应外界环境的信号刺激,自磷酸化其His残基,然后将磷酸基团转移到应答调控器的Asp残基上,通过这种His-Asp磷酸化传递来激活下游特异的信号输出^[18]^。除双/多组分系统外,pHis还参与磷酸化丙酮酸-糖磷酸转移酶系统(PTS),介导糖类以磷酸化的形式穿过细胞膜^[19]^。综上所述,pHis在原核生物和低等真核生物的生命过程中发挥重要调控作用。

pHis在哺乳动物中的生物学功能研究也取得了相应的进展,目前已发现两种pHis激酶(NDPK-A和NDPK-B)和3种磷酸酶(PHPT-1、LHPP和PGAM5)。此外,研究表明Histone H4可能具有pHis激酶活性^[20]^。pHis最重要的生物功能是通过激酶和磷酸酶可逆调控离子通道的活性^[16]^。Srivastava等^[21]^发现NDPK-B通过磷酸化钙激活钾通道KCa3.1358位的组氨酸来启动KCa3.1活性,从而促进Ca^2+^内流和T细胞的活化。而磷酸酶PHPT-1可直接与KCa3.1结合,去磷酸化H358位,从而对T细胞的活化进行负调控^[22]^。2016年,Panda等^[23]^发现PGAM5能够去磷酸化NDPK-B上的H118位,从而抑制KCa3.1的磷酸化,负调控T细胞的活化。与上述研究类似的是NDPK-B和PHPT-1通过介导TRPV5的H711位的磷酸化发挥重要调节作用^[24]^。此外,NDPK-B和PHPT-1通过可逆磷酸化G蛋白β1亚基上的H266来调控异三聚体G蛋白的活化^[25]^。2018年,Hindupur等^[26]^在mTOR驱动的肝细胞癌小鼠模型中发现NDPK-A和NDPK-B上调,而LHPP下调。LHPP的表达下调是肿瘤特异性的表现,进一步证明LHPP是重要的抑癌因子。LHPP在甲状腺癌、大肠癌、膀胱癌、宫颈癌和胰腺癌等肿瘤中均呈现下调,其高表达可以抑制肿瘤细胞的增殖,表达下调会促进肿瘤转移^[27]^。此外,LHPP表达可能与神经炎症有密切关系,可能调控神经退行性疾病的发生和发展。总之,哺乳动物pHis的生物功能研究还处于起步阶段,局限于几个重要的激酶和磷酸酶。 随着*N*-磷酸化蛋白质组学的发展,会发现更多具有活性的pHis蛋白质。

### 1.2 pArg的生物功能

pArg在原核生物中的生物功能研究已经逐渐得到阐释^[28]^,而在哺乳动物中的研究相对滞后。Clausen等^[29]^在2009年报道枯草芽孢杆菌蛋白质McsB是一种pArg激酶,其作用是调控基因转录的启动。当细菌受到热刺激时,McsB磷酸化转录因子CtsR的Arg62位由于电荷变化导致CtsR与clpC解离,进而启动clpC的基因转录过程。此外,蛋白质精氨酸磷酸化被证明是ClpCP蛋白酶体降解的标签,精氨酸磷酸化蛋白质可以被ClpCP蛋白酶体捕获降解,及时清理细胞内的受损蛋白及难以执行功能的蛋白质,在蛋白质量控制中起关键作用^[30]^。2012年,YwlE蛋白被鉴定为蛋白质pArg酯酶^[31]^。综上所述,pArg在细菌尤其是革兰氏阳性菌的生命过程中发挥重要作用。

## 2 蛋白质*N*-磷酸化修饰富集方法

近20年来蛋白质*O*-磷酸化修饰富集方法得到了长足进步,超过数万个*O*-磷酸化位点被规模化鉴定出来,规模化鉴定进一步促进了*O*-磷酸化生物功能的研究,如针对酪氨酸磷酸化的激酶抑制剂被开发用于肿瘤治疗。然而,*N*-磷酸化功能研究还处于起步阶段,主要原因是*N*-磷酸化修饰的化学性质不稳定,难以实现规模化鉴定。*N*-磷酸化修饰中P-N酰胺键水解吉布斯自由能较高,在酸和热条件下不稳定^[32]^。然而,目前针对*O*-磷酸化的富集方法需要强酸富集条件,并不适用于*N*-磷酸化富集。因此,亟需发展针对*N*-磷酸化修饰的富集方法,以实现其规模化鉴定。本文总结了近20年来*N*-磷酸化修饰富集方法进展,以供读者在研究其生物学功能时使用。

### 2.1 亲和富集法

基于抗体或蛋白结构域等的亲和富集法在*O*-磷酸化富集中得到广泛应用,根据富集原理,也可以用于非典型性磷酸化富集^[33]^。基于抗体的亲和富集法也是实现非典型性磷酸化富集的重要手段。研究表明抗pTyr抗体可以与pHis发生交叉反应,然而该抗体不能区分pHis和pTyr,限制了pHis的选择性富集^[34]^。研究表明可通过合成*N*-磷酸化氨基酸的稳定类似物作为抗原,通过动物的免疫反应获得*N*-磷酸化抗体。目前pHis抗体研究获得快速发展,已经有商品化抗体出售。如图2所示,本文总结了几种pHis类似物的分子结构。Muir等^[35]^合成了三唑类化合物(1)和(2),分别作为3-pHis和1-pHis的类似物。受到单独使用半抗原产生pTyr抗体工作的启示,Muir等^[36]^通过使用连接头将三唑基乙胺(3)与匙孔血蓝蛋白(KLH)结合来产生多克隆抗体。纯化的抗体可以用于富集已知的pHis肽段,但是与pTyr具有显著的交叉反应。将该多克隆抗体应用于*E. coli*裂解液中内源性pHis蛋白质的富集研究中,在甘油和甘露醇培养的*E. coli*中一共鉴定到16个内源性pHis位点,新发现醛醇脱氢酶(AdhE)和丙酮酸激酶(PykF)发生了pHis修饰^[37]^。然而,由于该抗体结合力有限,pHis位点的鉴定数目较少。随后,Kee等^[38]^报道了采用吡唑乙胺(4)作为3-pHis类似物制备第二代pHis抗体。密度泛函计算表明,吡唑类似物(4)与3-pHis结构和电子分布匹配,由此获得的多克隆抗体对pHis的响应灵敏度远高于pTyr。同时,Lilley等^[39]^利用吡唑氨基酸化合物(5)作为pHis类似物获得了类似的结果。2015年,Huntter等^[40]^制备出抗pHis的单克隆抗体。他们将(6)和(7)分别添加进中性肽库中形成两类肽段抗原,并结合KLH蛋白来引起免疫反应。获得的两种抗体能够分别特异性地识别3-pHis和1-pHis。利用该抗体在HeLa细胞中鉴定到786个潜在的pHis蛋白质,验证了pHis单克隆抗体具有优异的内源性pHis富集能力。

**图2:**
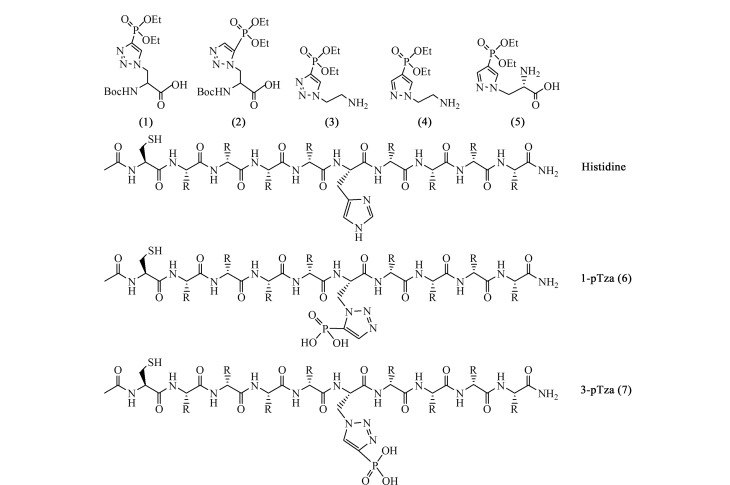
pHis类似物的结构

相对于pHis抗体的研究,pArg抗体的研究报道仍然较少。Clausen等^[41]^发现精氨酸激酶McsB在革兰氏阳性菌中发挥转录调节和蛋白质降解作用。采用噬菌体展示技术产生靶向pArg肽段的抗体。虽然这种抗体可用于重组蛋白的体外研究,但对pArg肽段的亲和力较低,难以实现富集研究。为获得亲和力较高的pArg特异性抗体,Thompson等^[42]^合成了酸稳定的PO_3_-脒和SO_3_-脒化合物作为pArg类似物,制备了不依赖序列的高亲和力抗pArg抗体,实现细胞裂解液中pArg蛋白ClpC和GroEL的检测。此外,赵玉芬等^[43]^也合成了类似的分子用于pArg抗体的制备。今后的研究需要进一步提高抗体的亲和力,通过在材料表面固载高亲和抗体研制功能富集材料,进而实现pArg肽段/蛋白质的选择性富集。Clausen等^[44]^同时开发了YwlE突变体,该突变体保留了对pArg的亲和性,可选择性地结合pArg蛋白。而赖氨酸磷酸化抗体研究仍处于空白状态,发展稳定类似物作为半抗原从原理上可行,期待有机合成技术的进步促进pLys抗体的制备和应用。

### 2.2 固定化金属离子色谱(IMAC)材料

在强酸条件下IMAC已成功用于*O*-磷酸化修饰富集,通过调节富集条件,IMAC也被尝试用于非典型性磷酸化富集研究^[45]^。2003年,Napper等^[46]^通过优化离子类型和富集pH值,证明Cu^2+^-IMAC在弱酸(pH 3.5)条件下能够选择性富集pHis肽段,材料富集性能有限,只能用于简单的肽段混合物,无法用于复杂生物样品的pHis规模化分析。最近,Lemeer等^[47]^发现部分pHis肽段在弱酸条件下结构保持稳定,前期研究结果证明Fe^3+^-IMAC在pH 2.3弱酸条件下对磷酸根具有优异的富集性能。通过结合这两点发现,他们将Fe^3+^-IMAC用于*E. coli*中pHis规模化鉴定,结合蛋白质沉淀和核酸去除策略去除干扰,总共鉴定出1447个*O*-磷酸化位点和135个pHis位点,组氨酸磷酸化位点约占总磷酸化位点数的10%,说明pHis在原核生物中含量和丰度较高,根据研究内容绘制了样品处理和流程图(图3)。利用该方法不仅能够鉴定出高丰度的代谢酶(约15000 copies/cell),低丰度的组氨酸激酶传感器arcB和dcu也能够得到鉴定(10~100 copies/cell)。作者进一步将该方法用于金黄色葡萄球菌中pArg位点鉴定,在耐甲氧西林金黄色葡萄球菌中鉴定出1062个pArg位点,发现磷酸酶Stp1可以增加pArg位点数目,但其本身不具备pArg激酶特性^[48]^。上述结果说明在弱酸条件下Fe^3+^-IMAC对*N*-磷酸化肽段具有较好的富集作用,但是弱酸条件难以适用于更加不稳定的pLys。

**图3:**
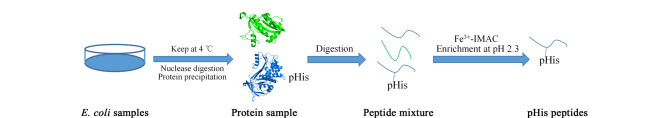
基于Fe^3+^-IMAC的弱酸环境富集pHis的实验流程和鉴定结果

近期秦伟捷等^[49]^利用二硫化钼纳米片作为基质材料,通过静电相互作用键合Ti^4+^离子制备功能二维IMAC材料(MoS_2_-Ti^4+^)。在弱酸(pH 3.0)富集条件下实现了HeLa细胞pHis规模化分析,共鉴定到159 pHis位点。研究结果进一步证明了在弱酸性条件下富集pHis的可行性,但是这类弱酸富集建立在损失鉴定覆盖度的基础上。发展用于*N*-磷酸化修饰高效富集的IIMAC材料仍然具有高度挑战。金属有机骨架材料(metal-organic frameworks, MOFs)可认为是一类特殊的IMAC材料。在MOF基底材料中,制备材料基底的有机骨架单元也是用于固定金属离子的配体,有机配体分子与金属离子交替配位形成周期性骨架结构,材料表面配位不饱和的金属离子提供了吸附磷酸化肽的活性位点^[50]^。该类材料具有大比表面积、超高孔隙率、易于功能化修饰以及良好的化学稳定性等优点,具有高效选择性富集磷酸根的潜力,在后续研究中可以尝试在中性条件下利用功能化MOFs材料高效富集*N*-磷酸化肽段。

### 2.3 Phos-Tag功能化材料

Phos-Tag是一类具有双二甲基吡啶胺-双锌离子(Ⅱ)复合物的金属配合物分子,Phos-Tag复合物中双Zn^2+^的空轨道可以与磷酸基团中氧原子的孤对电子配位,除配位外两者之间还存在静电引力,因此Phos-Tag可以识别和捕获具有磷酸基团的分子。Phos-Tag固定在聚丙烯酰胺凝胶电泳(SDS-PAGE)上已实现了磷酸化蛋白在凝胶上的分离和检测,同时也在肽段/蛋白质水平进行富集^[51]^。张丽华课题组^[52]^提出了在中性条件下采用Phos-Tag功能材料富集*N*-磷酸化肽段的策略(图4)。由于P-N键比P-O键具有更强的给电子能力,等温滴定量热结果显示Phos-Tag与*N*-磷酸化肽段之间的解离常数达μmol/L,证明Phos-Tag具备富集复杂生物样品中*N*-磷酸化肽段的能力。课题组随后制备Phos-Tag修饰的亚二微米核壳硅球(SiO_2_@DpaZn),构建了在线On-Tip富集系统,该系统具备在中性条件下识别磷酸根以及快速富集的优点。采用该系统可实现*N*-磷酸化肽段的上样、清洗和富集,处理时间比离线富集方式缩短了3倍,回收率提高了4倍。富集机理研究表明配位作用为材料与肽段之间的主要作用力,静电和亲水相互作用为次要作用力,机理研究为后续材料设计提供了理论指导。同时利用该材料在HeLa细胞中鉴定了超过3000个*N*-磷酸化位点,研究结果显示*N*-磷酸化蛋白质在哺乳动物细胞生物代谢、免疫反应等通路明显富集,与ATP结合、RNA结合、核苷酸结合和蛋白激酶结合等功能密切相关。进一步发现*N*-磷酸化修饰位点周围亮氨酸高度富集,这为*N*-磷酸化蛋白质功能和激酶/酯酶的底物发现提供了新的线索。该策略同样适用于在*N*-磷酸化蛋白质水平的富集研究^[53,54]^。作者进一步利用该方法发现5xFAD模型鼠脑蛋白质*N*-磷酸化修饰升高,尤其是骨架蛋白^[55]^。但硅球作为基质存在被缓冲液刻蚀的情况,会引起质谱灵敏度降低,亟需发展稳定性好、固载量大、亲水性强的基质用于Phos-Tag固载。

**图4:**
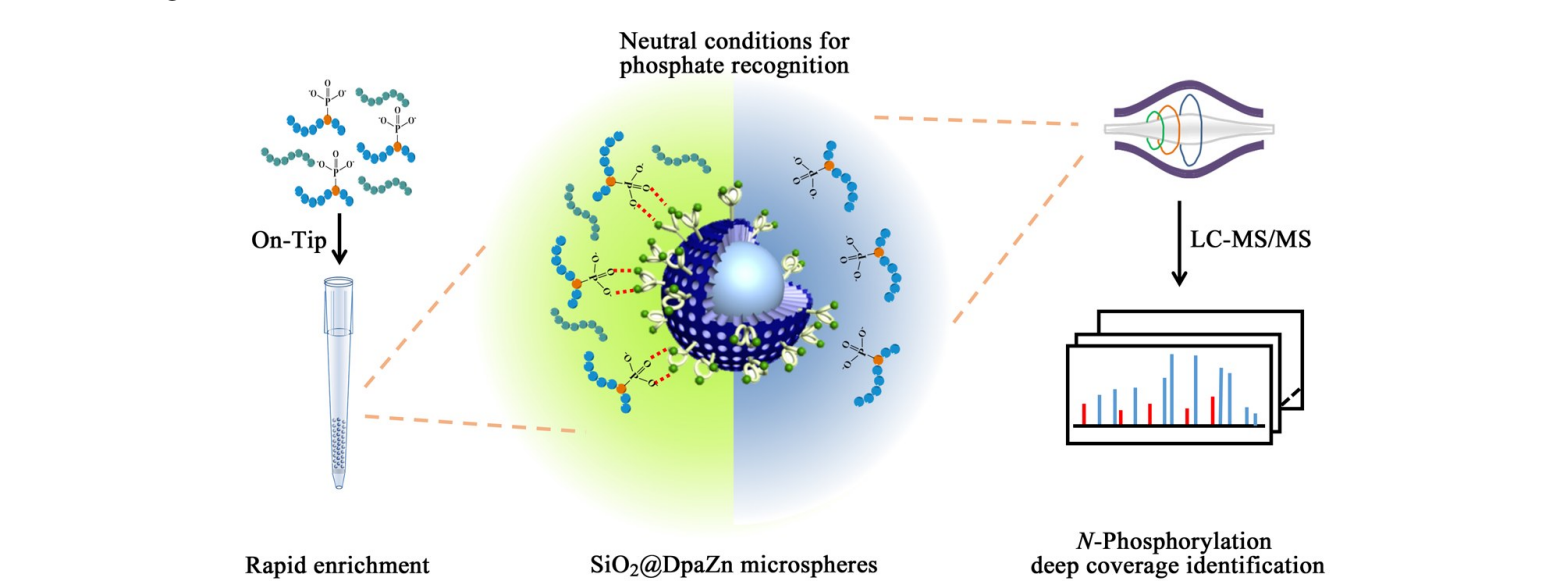
基于Phos-Tag功能化亚二微米核壳硅球的中性条件下*N*-磷酸化肽段富集

### 2.4 金属氧化物色谱(MOAC)

在强酸条件下MOAC已成功用于*O*-磷酸化肽段富集。通过调节富集条件,MOAC也被尝试用于非典型性磷酸化富集研究^[56]^。Clausen等^[57]^利用TiO_2_在弱酸性(pH 4.0)的富集溶液系统下,在酯酶突变型、热刺激的枯草芽孢杆菌中鉴定到134个蛋白质上的217个pArg位点,通过标准肽段验证pArg在弱酸条件下的稳定性。在弱酸性富集条件下,金属氧化物和磷酸根之间结合力较弱,高丰度的非磷酸化肽严重干扰磷酸化肽的鉴定,因此鉴定到的pArg鉴定数目较少,其原理同IMAC材料在弱酸性条件下富集非典型性磷酸化肽类似,该方法的选择性和回收率都有待提高。近期,Schmitz等^[58]^利用TiO_2_在pH 4.0条件下富集pArg位点,验证结核分歧杆菌中pArg的广泛存在。赵玉芬等^[59]^在Jurkat细胞中鉴定出121个pHis位点,152个pArg位点,153个pLys位点,这些位点对应的蛋白质主要参与DNA结合,与HeLa细胞中鉴定的结果类似,结果说明*N*-磷酸化修饰可能在细胞核中富集。根据IMAC研究结果,可将富集条件进一步降低到pH 2.3,或可进一步提高pHis的鉴定覆盖度。弱酸富集是建立在牺牲覆盖度的基础上,研究趋势是发展结合力更强的MOAC以实现中性条件下*N*-磷酸化肽段的选择性。

### 2.5 化学标记法

利用磷酸化类似物作为半抗原制备组氨酸磷酸化和精氨酸磷酸化抗体,然而由于缺乏赖氨酸磷酸化类似物的合成方法,无法获得抗pLys抗体。因此,目前尚无针对pLys肽段的分析方法。张丽华等^[60,61]^根据pLys肽段在酸性条件下易水解的特点,利用赖氨酸磷酸化肽段与赖氨酸去磷酸化肽段在反相色谱柱上保留差异,并结合二甲基化标记来实现pLys肽段的特异性分析。富集流程示意图如图5所示,首先利用二甲基化(轻标)封闭肽段混合物N端和赖氨酸侧链氨基;然后对肽段进行第一维高pH反相分级,将获得的分级产物进行酸化使赖氨酸磷酸化水解;最后对于酸化后的分级产物进行第二维高pH反相分级,由于去磷酸化肽段疏水性增强,导致在反相色谱柱上保留增强,收集保留时间后移部分,对收集的去磷酸化肽段用二甲基(重标),具有一个轻标和一个重标的赖氨酸位点即发生了磷酸化修饰。利用该方法实现了在100000倍干扰下赖氨酸磷酸化肽段的选择性富集和鉴定,从*E. coli*样品中鉴定到11条赖氨酸磷酸化肽段,对应10个蛋白质,采用疏水基团代替二甲基重标,将*E. coli*中的赖氨酸磷酸化位点的鉴定数目提高到39个。上述研究为赖氨酸磷酸化肽段提供了一种新的分析方法,为探索赖氨酸磷酸化蛋白质的生物学功能奠定了基础。这种标记存在的问题是赖氨酸不仅仅存在不稳定的*N*-磷酸化修饰,如果存在其他酸不稳定修饰,会导致鉴定的假阳性。近期应万涛等^[62]^首先利用在pH 2.7的弱酸性溶液中pHis肽相对于His肽的低电荷状态,使用强阳离子交换(SCX)色谱法区分pHis肽和His肽。其次,采用Cu-IDA技术对pHis肽选择性富集。最后,引入稳定同位素二甲基标记技术,保证pHis肽的高可靠识别。利用该整合策略,从HeLa细胞中鉴定到563条不同的pHis肽。肽段序列分析表明,pHis肽具有一致的基序HxxK并偏好疏水氨基酸,覆盖了以往几种研究的结果。综上所述,化学标记提供了一种分离富集某一类*N*-磷酸化肽段的手段,但难以实现3类*N*-磷酸化肽段的普适性富集。

**图5:**
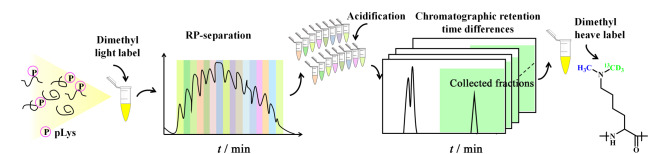
对角线色谱结合二甲基化标记策略富集pLys流程

### 2.6 蛋白质*N*-磷酸化富集存在的问题

与非磷酸化蛋白质相比,磷酸化蛋白质在细胞或组织内的丰度低,在质谱鉴定过程中信号非常容易受到高丰度的非磷酸肽段的抑制;蛋白质磷酸化和去磷酸化是可逆的动态变化过程,同一个磷酸化蛋白质在不同时刻的绝对数量在不断变化,甚至在富集时刻从磷酸化状态变成非磷酸化状态,导致难以精确鉴定;细胞或组织内存在多种磷酸酶,在对蛋白质样品进行处理时,容易引起样品的去磷酸化反应,从而可能造成磷酸化样品损失。这些普适性的因素也会造成*N*-磷酸化修饰鉴定困难。此外,还有其他因素影响*N*-磷酸化鉴定。(1)NanoLC-MS/MS分离阶段需要用甲酸作为添加剂调节分离能力,酸性分离条件容易导致*N*-磷酸化水解;(2)现在富集流程比较繁琐,而且大部分是离线操作,容易导致富集回收率较低;(3)当前仍旧缺乏统一的搜库检索参数,搜库结果容易出现假阳性,尤其容易将*O*-磷酸化误匹配成*N*-磷酸化;(4)现有富集方法鉴定的*N*-磷酸化位点重叠率较少,一方面由于*N*-磷酸化高度动态变化,另一方面是由于每种方法的识别机理不同;(5)磷酸化性质类似,难以实现*N*-磷酸化选择性富集。

## 3 总结和展望

本文总结了近20年来蛋白质*N*-磷酸化修饰富集方法的研究进展,亲和抗体、IMAC、Phos-Tag功能化材料、MOAC、化学标记法已经被开发用于*N*-磷酸化修饰的富集研究。通过这些方法已经有超过数千个*N*-磷酸化位点被鉴定到,这些方法可用于原核生物和哺乳动物*N*-磷酸化位点的发现。利用已经发现的位点,研究人员建立了*N*-磷酸化位点数据库^[63]^。这些研究极大地促进了*N*-磷酸化的研究。尽管*N*-磷酸化富集方法研究取得了一定进展,但相对于*O*-磷酸化富集方法,*N*-磷酸化富集方法发展依旧滞后。随着生物技术、化学合成和材料科学的发展,一些新富集策略可尝试用于*N*-磷酸化修饰的富集研究。

智能聚合物基材料通过外部物理、化学或生物刺激可逆地改变其结构和功能,可实现对磷酸化肽高度可控的吸附和脱附,实现高选择性富集。一方面,智能聚合物基材料的响应变化包括材料疏水性的增加或减少、形状和形貌的改变、表面电荷的重新分布以及亲和配体的暴露或隐藏等特性。这些特性使得磷酸化肽段和智能聚合物基材料之间的亲和力可以通过简单改变外部条件(如温度、pH值、溶剂极性和生物分子等)而改变,进而实现更可控和更智能的精细调节。另一方面,智能聚合物基材料为集成功能模块提供了便捷的可扩展平台,例如特定的识别组件,显著提高磷酸化肽段的分离选择性^[64]^。如杨海波等^[65]^通过结合金属配位自组装和后修饰的聚合反应,制备了一种新型的以有机铂金属大环为核心骨架的超分子聚合物。该超分子聚合物同时包含氢键和静电相互作用等多种磷酸根识别位点,因此对磷酸化肽的结合具有较高的选择性,金属环骨架上的多个正电荷使聚合物层与层之间通过静电排斥彼此分离,从而形成稳定且易于转移的二维超分子聚合物。因此,有利于增加磷酸化肽与聚合物材料间的接触面积,从而实现在水溶液中快速高效地捕获磷酸化肽。通过简单的水洗和离心即可方便地除去非磷酸化肽。同时,得益于聚合物核心骨架独特的刺激响应性能以及与磷酸化肽在响应前后结合能力的巨大差异,该智能超分子聚合物可以在中性条件下实现对磷酸化肽有效的洗脱与释放。研究结果展现了对*N*-磷酸化肽段高效的富集潜力。

基于色谱的富集方法是实现*N*-磷酸化修饰高效富集的重要手段,Eyers等^[11]^利用强阴离子色谱作为富集手段,通过调节富集pH值,在中性条件下实现了*N*-磷酸化肽段的高效富集。类似的原理,发展亲水相互作用色谱,利用亲水固定相和*N*-磷酸化肽段之间亲水作用,也可在中性条件下实现*N*-磷酸化肽段的有效富集。另外可以制备分子印迹聚合物,虽然难以实现*N*-磷酸化肽段/蛋白质规模化富集,但是适用于单个*N*-磷酸化肽段/蛋白质的富集,如制备NDPK-B的分子印迹聚合物,利用化学性质和结构匹配实现NDPK-B的选择性富集。
